# Soil microbial communities in dry and moist tropical forests exhibit distinct shifts in community composition but not diversity with succession

**DOI:** 10.1128/spectrum.01931-24

**Published:** 2025-02-04

**Authors:** Kristin Saltonstall, Michiel van Breugel, Wayra Navia, Hilda Castillo, Jefferson S. Hall

**Affiliations:** 1 Smithsonian Tropical Research Institute, Panama City, Republic of Panamá; 2 Department of Geography, National University of Singapore, Singapore, Singapore; 3 Yale-NUS College, Singapore, Singapore; 4 ForestGEO, Smithsonian Tropical Research Institute, Panama City, Republic of Panamá, USA; University of Mississippi, University, Mississippi, USA

**Keywords:** Panama, bacteria, fungi, AMF, plant pathogens, nitrogen-fixing bacteria, pasture, secondary forest, metabarcoding

## Abstract

Soil microbial communities are integral to ecosystem function but our understanding of how they respond to secondary succession in fragmented landscapes is limited, particularly in tropical dry forests. We used DNA metabarcoding to evaluate successional changes in soil bacteria and fungi, comparing land managed for cattle, young, and older secondary forests at moist and dry sites in the Republic of Panama. We highlight key functional groups of microbes that interact with plants, including arbuscular mycorrhizal fungi (AMF), nitrogen-fixing bacteria, and plant pathogenic fungi. Plant diversity was higher at the moist site and increased with succession as the plant communities changed at both sites. By contrast, bacterial diversity was similar across sites and successional stages, and while overall fungal diversity was higher at the moist site, it also showed no changes with succession at either site. However, microbial community composition did change, with pastures and older forests having distinct bacterial and fungal communities and young secondary forests often displaying transitional ones. Functional groups of microbes showed contrasting patterns between sites, with the dry forest having a higher diversity of Nitrogen-fixing bacteria despite lower densities of legumes, higher diversity and different communities of AMF, and a much lower incidence of putative fungal plant pathogens than the moist site. Our findings highlight the importance of looking at aboveground and belowground effects together and demonstrate that predictions generated for soil microbes in moist tropical forests may not apply to dry forests. These results may also inform the restoration of climate-resilient forests.

**IMPORTANCE:**

Secondary forests are important components of neotropical landscapes and soil microbes help to shape these forests and the ecosystem services that they provide. This study demonstrates that soil microbial communities in moist and dry tropical forests can recover and reassemble after only 20 years of natural succession following the removal of cattle. However, successional patterns that are seen in the plant community are not always seen belowground. These patterns were more predictable at the moist than the dry site where the patchiness of the landscape likely restricts dispersal of both plants and soil microbes. We highlight the importance of preserving remaining tropical dry forests as they host unique microbial biodiversity that may help forests respond to drought conditions. As community shifts in soil microbes influence plant establishment, forest productivity, and other aspects of ecosystem functioning during the succession of tropical forest communities, our results can inform the restoration of climate-resilient forests.

## INTRODUCTION

Restoration of degraded lands seeks to regain ecosystem function and restore ecosystem services and biodiversity, a complex undertaking. Aboveground-belowground feedbacks are important in the processes of forest recovery and succession but our knowledge of whether or not plant and soil microbial communities go through coordinated shifts during the processes of succession and ecosystem recovery in tropical regions is limited (1–3). It is clear that successional trajectories of tropical plant communities may vary widely depending on site conditions (4). A better understanding of the distribution of soil microbial communities in natural ecosystems and the environmental factors that influence them will aid in forecasting their ability to continue to provide valuable services to these forests under projected climate and land-use change scenarios.

Processes of land use that change soil physical, chemical, and microbiological processes are well studied. Livestock management can alter the physical and biological properties of soil (5) and abiotic conditions change as forests establish and succession proceeds. This can include decreases in light levels, increases in relative humidity and soil moisture levels, and transition of plant communities from fast-growing, light-demanding species to slower-growing, shade-tolerant species (6, 7). Microbial succession, like plant succession, is influenced by resource limitation, environmental stress, biological interactions, and historical effects (8, 9). However, differences in phylogenetic diversity, metabolic versatility, reproductive rate, and dormancy between plants and microbes may lead to distinct mechanisms driving the recovery of soil microbiota during forest succession. Many studies have identified driving factors, such as soil properties and chemistry (10–12) and climate (12–17), as being important factors structuring belowground microbial communities, but others have shown that patterns of belowground diversity do not necessarily respond consistently to abiotic parameters (1, 18). Much less is known about how biotic interactions influence this process. There have been conflicting reports as to the importance of plant identity and plant species composition in predicting soil microbial communities (10, 19–22) although plants clearly impact physical microhabitats and food supplies for soil microbes (9, 23). As biotic interactions are inherently complicated and abiotic factors vary spatially, studies at sites with uniform abiotic conditions can enhance our understanding of the community assembly of soil bacteria and fungi.

Soil microbes interact with plants, either directly or indirectly and both positively and negatively (24–26). Certain bacteria and fungi can have a broad range of positive interactions with plants, including nitrogen fixation, nutrient and water uptake, protection against pathogens and pests, and inducing tolerance to abiotic stresses (27, 28). The largest natural input of nitrogen to tropical forests is thought to derive from leguminous trees that can fix atmospheric nitrogen through their symbiosis with rhizobia bacteria (29–31). Most trees in Neotropical forests also associate with arbuscular mycorrhizal fungi (Glomeromycota; hereafter AMF) which have been shown to enhance nutrient and water uptake, thus increasing host fitness by providing stress resistance and facilitating growth (32). Plant pathogens may be generalists or specialists and negatively impact plant survivorship and growth, yet are broadly considered to be important for the maintenance of tropical forest tree diversity (33, 34).

Functional groups of microbes may respond differently than total communities to the abiotic conditions in the soil, with low nutrient soils increasing diversity and abundances of organisms such as arbuscular mycorrhizal fungi (20, 35), oligotrophic bacteria, and nitrogen (N_2_)-fixing bacteria (36, 37). Soil pH, temperature, and precipitation have also been shown to structure bacterial, AMF, and plant pathogen communities (15, 38–40). However, as many microbes disperse passively, the establishment and recovery of these plant-associated microbial communities during succession could be highly dependent on the surrounding land use matrix and the intensity of human impacts (1, 41, 42).

Here we identify key differences in the distribution of soil microbes in seasonal moist and dry tropical forests in the Republic of Panama that are undergoing succession. We used DNA metabarcoding to explore how the diversity and composition of soil bacterial and fungal communities change across three successional stages (active cattle pastures, young secondary forests [YSF], and older secondary forests [OSF]), with an emphasis on specific functional groups including AMF, N_2_-fixing bacteria, and plant fungal pathogens. Soil chemistry is remarkably similar within each landscape (hereafter referred to as site), and all plots are located such that differences in climatic influences are minimal (43, 44), allowing us to make inferences about the influence of aboveground changes in vegetation on soil microbial communities during forest succession. As the two sites have different annual rainfall patterns, soil chemistry, and distinct plant communities, we expected these soil microbial communities to be different between sites and focus on within-site changes while highlighting key differences between moist and dry forests that may have implications for future restoration efforts.

## RESULTS

### Environmental differences between sites

Our two study sites differ in a variety of factors that may influence soil microbial communities. The moist site (Agua Salud) experiences higher rainfall with a shorter dry season than the dry site (Los Santos). Soil chemistry also differs, with an average soil pH at the moist site of 5.0 versus 6.0 at the dry site. Many soil nutrients, including Ca, Mg, P, and Zn, had significantly higher concentrations at the dry site, whereas Al and Mn were higher at the moist site. However, within each site, most soil properties, including pH (moist: R^2^ <0.01, *P* > 0.05; dry: R^2^ = 0.04 , *P* > 0.05), were homogeneous across successional stages ( Table S1).

**Fig 1:**
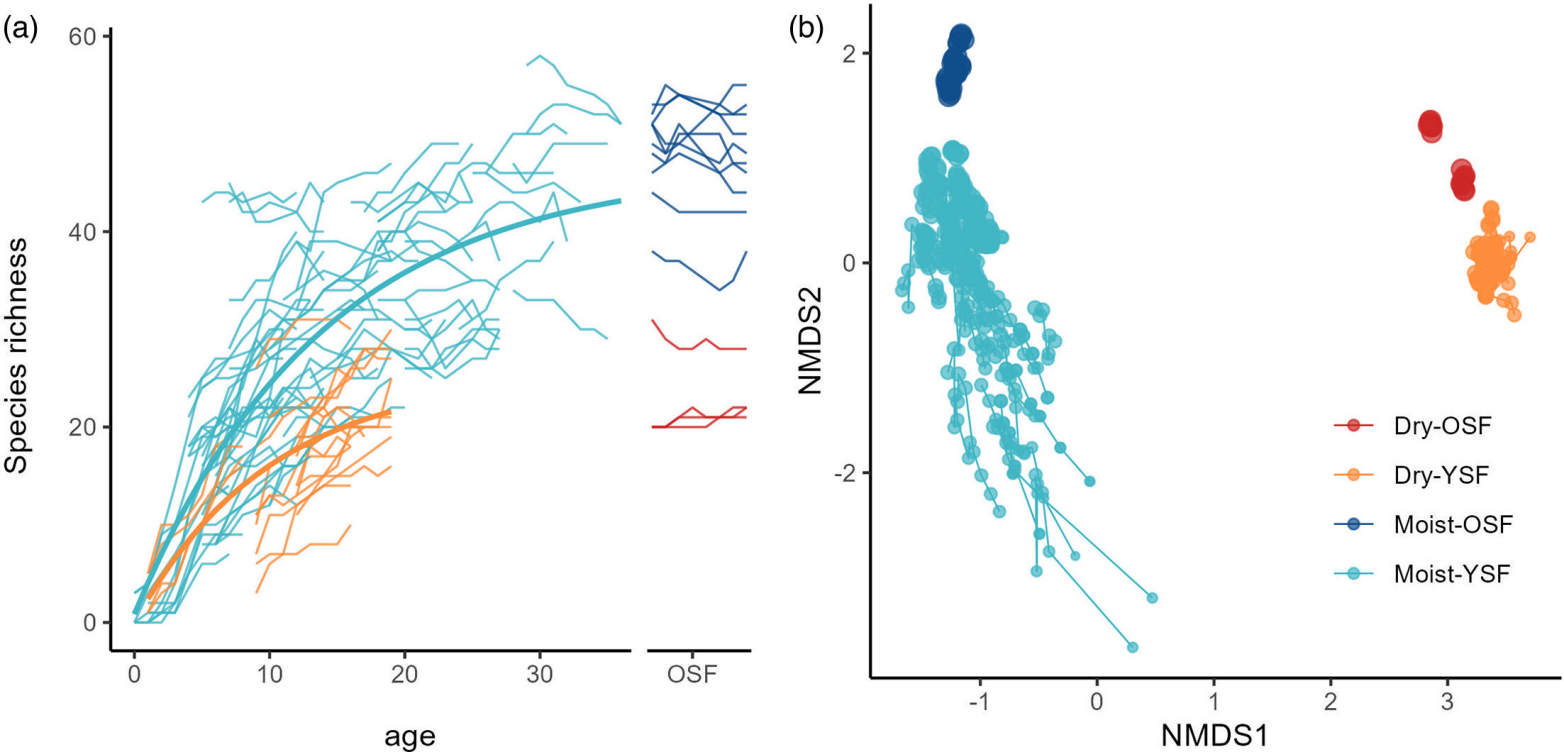
Successional patterns in tree species richness and species composition across YSF and OSF in a moist and dry tropical landscape. The age of the OSF plots was unknown but all are >80 years. (a) Species richness of stems ≥5 cm DBH. Thin lines plot the changes in species richness of individual plots over an 8-year study period and across each secondary forest network. Asymptotic non-linear regression models were fitted to the data to visualize general successional trajectories. (b) Ordination biplot of the first two axes of a non‐metric multidimensional ordination based on Ružička dissimilarity. Dots connected by lines represent individual plots and dot size is proportional to the stand basal area.

Tree species composition differed strongly between the moist and dry plant communities with only 93 shared between sites (13.2%), out of a combined species pool of 704 species. Tree species richness increased (Fig. 1a) and species composition shifted (Fig. 1b) over the course of succession, with much higher diversities at the moist site. Only 7 out of a total of 35 legume species (20%) were found at both sites. The average percent basal area of legumes was three times higher in YSF than OSF at both sites, but their overall relative abundance was significantly higher at the moist site than the dry site (moist: 3.8 ± 1.8% vs dry: 1.8 ± 0.8%, *P* < 0.05). The relative abundance of deciduous species was much higher at the dry site than at the moist site (69.0 ± 12.6% vs 13.5 ± 6.3%), while the opposite was true for semi-deciduous species (21.0 ± 8.5% vs 47.8 ± 6.8%). All pastures were dominated by grasses (Poaceae), especially *Ischaemum ciliare* Retz. at the moist site and *Hyparrhenia rufa* (Nees) Stapf and *Megathyrsus maximus* Jacq. at the dry site.

**Fig 2:**
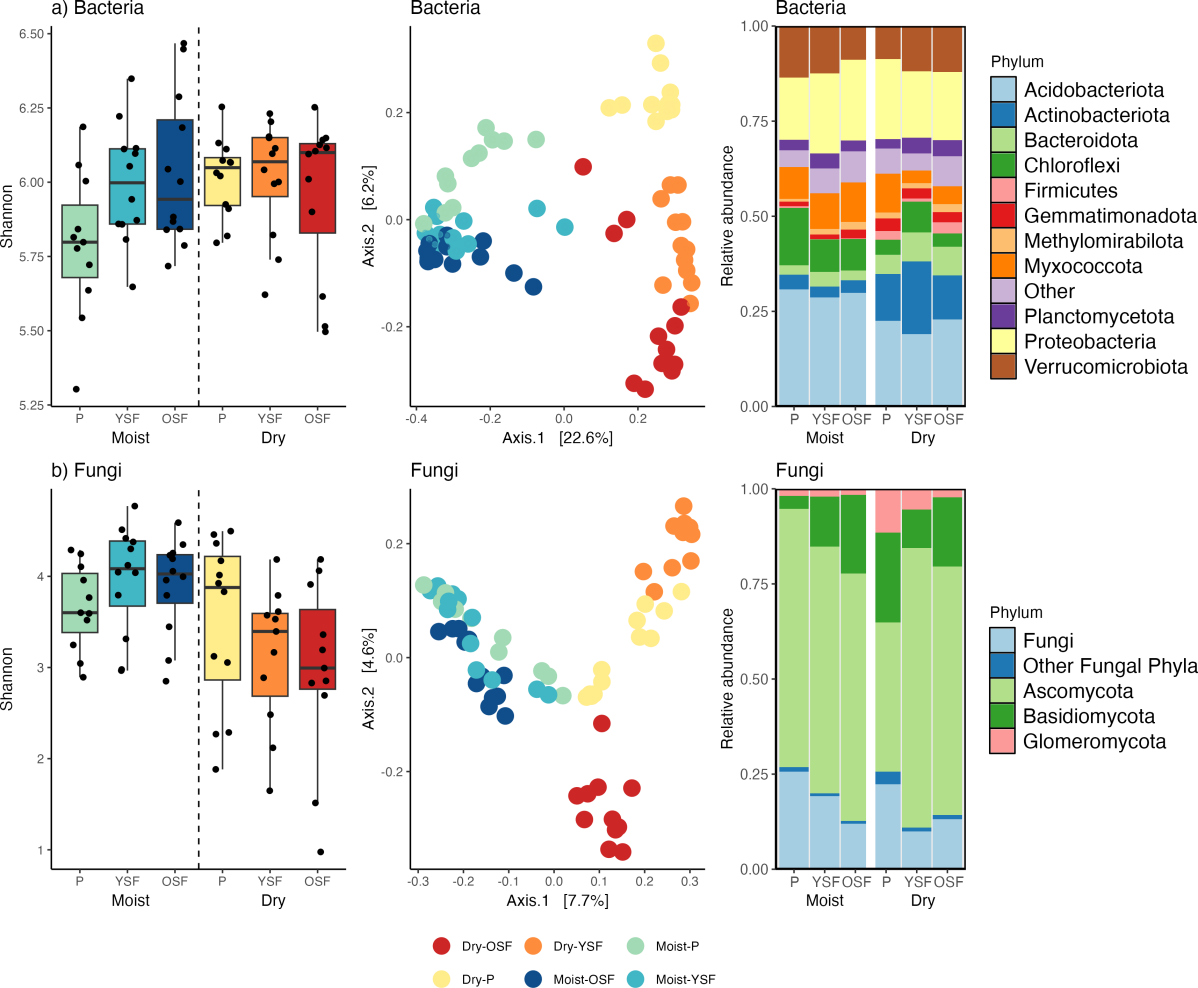
Successional patterns in species richness and community composition in soil bacteria and fungi across succession in a moist and a dry tropical landscape**.** Shannon diversity, principle coordinates analysis based on Bray-Curtis dissimilarity of Hellinger transformed community data, and relative abundance of (a) bacteria and (b) fungi. Points represent individual soil samples and numbers in brackets along the axes indicate the percent of total variation explained by the axis. The center line of each box plot represents the median, the lower, and upper hinges represent the first and third quartiles and the whiskers represent +1.5 the interquartile range. P = Pasture, YSF = Young secondary forest, OSF = Old secondary forest.

Microbial communities also differed between the moist and dry sites. Although no significant differences in Shannon diversity of the total bacteria communities were found between the moist and dry forests (Fig. 2a), nearly all phyla of bacteria showed significant differences in relative abundance between the two sites (Fig. 2a; Table S1a). Major community differences were largely driven by Acidobacteriota (especially the Orders Acidobacteriales, Subgroups 2 and 5, and Vicinamibacterales) and Actinobacteria (particularly the Orders Frankiales, Gaiellales, and Solirubrobaerales; Fig. S1a and b). Both Shannon and Inverse Simpson diversity of fungi was higher in our moist forest as compared to our dry site (*P* < 0.05, Fig. 2b; Table S2b). Ascomycota dominated both sites, but overall community composition differed between sites, with the moist forest having higher diversity and relative abundance of Ascomycota and Unidentified Fungi and the dry site having higher diversity and relative abundance of Glomeromycota (LDA 4.0, *P* < 0.05; Fig. 2B, Fig. S2a through c). In general, microbial communities showed higher dissimilarity across successional stages at the dry site than at the moist site (Fig. 2; Fig. S1 and S2 ).

### Successional patterns in moist forest

No significant differences in total bacterial or fungal diversity (Shannon, Inverse Simpson) were detected between successional stages at the moist forest (Fig. 2a). However, YSF and OSF had a higher diversity of several dominant phyla, including Bacteroidota, Proteobacteria, and Verrucomicrobiota, than Pastures (Fig. S1). Fungal phyla showed few differences in diversity between successional stages (Fig.S2).

Community composition shifted with succession for both bacteria and fungi at the moist site and explained 14 and 13% of the variation in communities (Fig. 2; Table S4a). PCoA ordinations showed communities of bacteria in Pastures to be distinct from both YSF and OSF (pairwise adonis: *P* < 0.001), which had similar composition (Fig. 2a). These differences in the pasture community were largely driven by members of the Acidobacteriota, Proteobacteria, and Verrucomicrobiota (Fig. S1). Total fungal communities in Pastures also differed from OSF (*P* < 0.001), but not YSF (Fig. 2b), although the community of Glomeromycota in Pastures (as identified with ITS) was significantly different from both YSF and OSF and more similar to the Glomeromycota communities at the dry site (*P* < 0.001, Fig. S2c). Pairwise tests for homogeneity of multivariate dispersions confirm that these patterns in both bacterial and fungal communities are not due to differences in group dispersions (PERMUTEST, *P* > 0.05).

**Fig 3:**
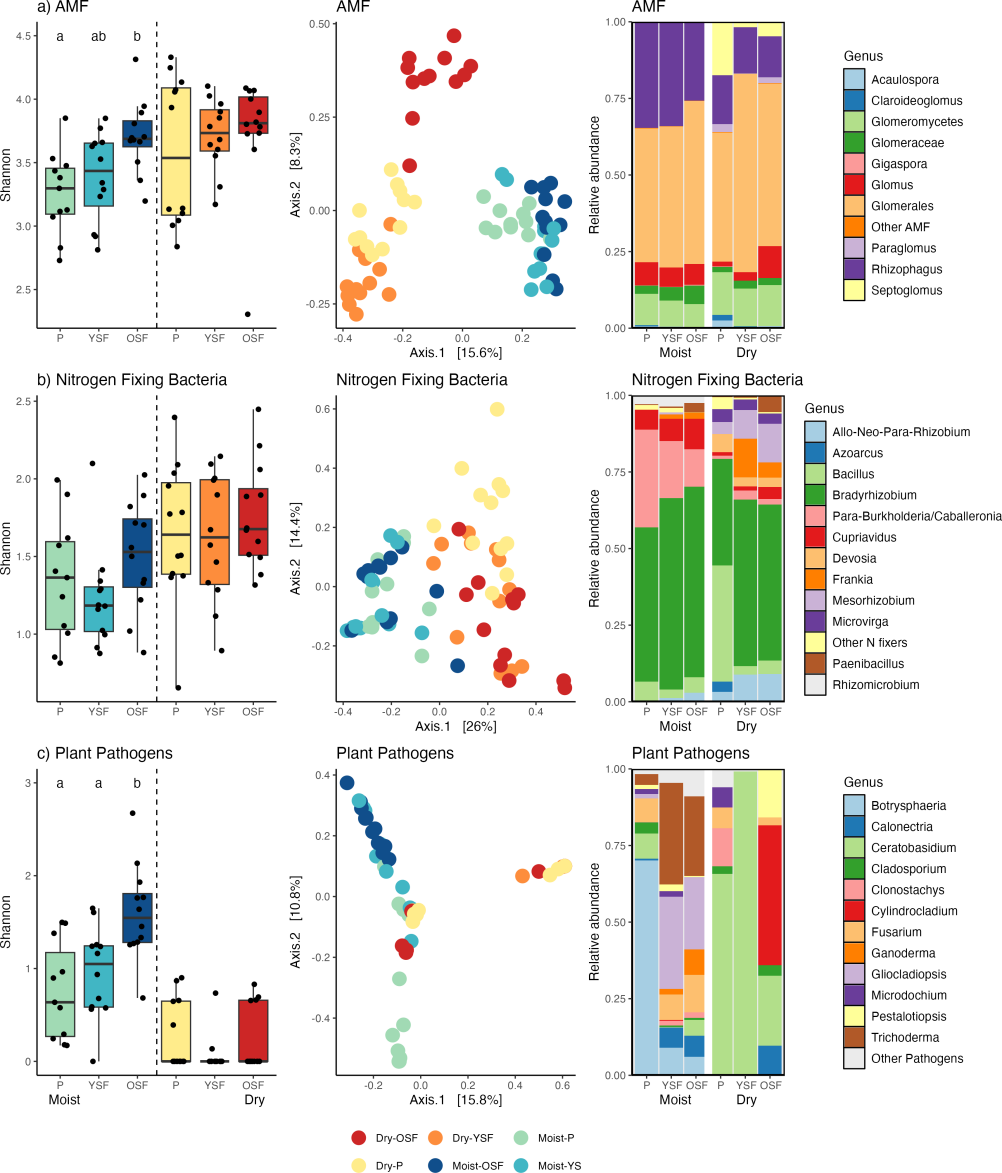
Functional groups of microbes respond to succession in moist and dry tropical landscapes. Shannon diversity of the total communities, Principle coordinates analysis based on Bray-Curtis dissimilarity of Hellinger transformed community data, and Relative Abundance of (a) AMF, (b) N_2_-fixing bacteria communities, and (c) plant pathogenic fungi across successional stages. AMF communities were characterized with 18S metabarcoding; N_2_-fixing bacteria data were subset from rarefied total bacterial communities (16S) and putative plant pathogens were subset from the rarefied total fungal communities (ITS). All communities were significantly different between sites (adonis, *P* < 0.001). Points in the PCoA plots represent individual soil samples and numbers in brackets along the axes indicate the percent of total variation explained by the axis. P = Pasture, YSF = Young secondary forest, OSF = old secondary forest.

Functional groups of microbes showed a stronger response to succession than soil nutrients at the moist site (Table S4). OSF had higher AMF diversity than Pastures and YSF (*P* < 0.05) and Pastures had unique communities (PERMANOVA, *P* < 0.05). The PCoA ordination suggests a transition in the community from Pastures to OSF, with YSF showing similarities to both (Fig. 3a). Of 27 AMF ASVs showing differential abundance (LDA 4.0, *P* < 0.05), 10 were enriched in Pastures and 17 were enriched in YSF and OSF. *Rhizophagus* was the most common genus and had a lower relative abundance in OSF than Pastures and YSF (Fig. 3a; Table S2c). AMF communities varied with soil pH, P, Cu, and Mn at the moist site, although the successional stage explained the most variation (Table S4a).

The N_2_-fixer community was similar across all successional stages and tended to be dominated by only three genera (*Bradyrhizobium*, *Burkholderia-Caballeronia-Paraburkholderia*, and *Cupriavidus*). Pastures tended to have lower relative abundances of *Bradyrhizobium* and had more Burkholderiaceae and *Bacillus* and different community composition than forested plots. Although there were more leguminous trees in YSF than in OSF, there were no differences in the diversity of N_2_-fixing bacteria between the two forest age classes. *Frankia* was only found in forested plots (Fig. 3b; Table S3a). Soil pH, P, and Cu likely influence N_2_-fixer communities although the successional stage explained the most variation (Table S4a)

The relative abundance and diversity of pathogenic fungi were higher at the moist site, and all samples contained identified sequences from these genera. In addition, the relative abundance of most pathogens was higher in forested plots than in Pastures (Fig. 3c; Table S3b). These included ASVs in the genera *Calonectria*, *Fusarium*, *Gliocladiopsis*, and *Trichoderma*, three of which were more abundant in YSF and OSF than Pastures (LDA 4.0, *P* < 0.05). By contrast, Pastures were dominated by *Botryosphaeria* (LDA 4.0, *P* < 0.05) and *Rhizoctonia* was only found in Pastures. OSF had higher diversity than Pastures and YSF although the community composition of YSF was similar to OSF (Fig. 3c). While it is commonly isolated from plant leaves and diseased tissues in moist forests of Panama (33), *Colletotrichum/Glomerella* was not detected.

### Successional patterns in dry forest

As seen at the moist site, no significant differences in total bacterial or fungal diversity were detected between successional stages at the dry site (Fig. 2a). However, YSF and OSF had a higher diversity of Bacteroidota and Verrucomicrobiota than Pastures (*P* < 0.0.5; Fig. S1). When identified with the general fungal primers (ITS1), both the overall relative abundance and the diversity of ASVs of Glomeromycota (AMF) were significantly higher at our dry site (1.9% ± 0.02% moist vs 6.9% ± 4.7% dry, Table S2b; Fig. S2c).

The successional stage had a strong influence on both bacteria and fungi at the dry site and explained 20 and 16% of the variation in these communities (Table S4b). PCoA ordinations showed the communities of each successional stage to be unique (PERMANOVA, *P* < 0.001, ANOSIM, *P* < 0.05), with clear separation along axis 2. Pasture bacteria were distinct from YSF and OSF (Fig. 2a) and fungal communities in forested plots showed no overlap (Fig. 2b). YSF communities displayed lower dispersion for both bacteria and fungi (PERMUTEST, *P* < 0.05) suggesting higher homogeneity in this successional stage across plots. Differences in the fungal communities were seen in all phyla (Fig S2).

Alpha diversity of functional groups of microbes also showed no significant response to succession at the dry site (Fig. 3a through c). All three successional stages had distinct AMF communities (Fig. 3a), likely driven by genera in the family Glomeraceae showing different patterns of abundance in each stage (Table S4b). Soil pH and P did not correlate with AMF community structure (*P* > 0.05; Table S4b). Diversity was more variable between samples in Pastures than forested plots (Fig. 3a), each successional stage had several ASVs associated with it, and YSF shared differentially abundant ASVs with both Pastures and OSF (LDA 4.0, *P* < 0.05).

Although the relative abundance of leguminous trees was only half that of the moist site, the overall diversity and relative abundance of N_2_-fixing bacteria was higher at our dry site, particularly within the family Rhizobiaceae. Successional stage explained the most variation in these communities, although pH was also important (Table S4b), and Pastures had different communities than forested plots (pairwise adonis *P* < 0.05, Fig. 3b). No differences in diversity were seen between successional stages and, similar to the moist site, the N_2_-fixer community was dominated by *Bradyrhizobium*. However, *Bacillus*, *Mezorhizobium*, *Microvirga*, and *Allo/Neo/Para-Rhizobium* taxa were also prevalent and likely drove the community differences in Pastures from other successional stages. *Frankia* was again found only in forested plots but had higher relative abundance and more ASV diversity than the moist site (10 vs 3 observed ASVs; Table S3a).

Plant pathogens, as defined in our study, were extremely rare at our dry site with 25% of the samples having no pathogen sequences and 39% with only a single ASV. Overall diversity and relative abundance of these putative pathogens was much lower than the moist site, with only 10 of the 20 included genera detected (Table S3b). Although no differences in alpha or community diversity were seen between successional stages (Fig. 3c), YSF plots were most likely to lack pathogen sequences. *Ceratobasidium* was the most common genus, particularly in Pastures and YSF where it was the dominant pathogen. *Cylindrocladium* and *Pestalotiopsis* were common in OSF. As seen in the moist site, *Rhizoctonia* was only found in Pastures (Table S3b).

## DISCUSSION

Our study shows shifts in community composition of soil microbes after only 20 years of forest succession following cattle removal, with clear differences between dry and moist Neotropical forests and among functional groups of microbes. We expected to find gradual increases in microbial diversity and shifts in community composition between successional stages that mirrored changes in the plant communities but our results did not always show this. While total bacterial and fungal communities changed as forest succession progressed with distinct communities between pastures and forested plots, their diversity remained constant. This turnover in taxa but not overall diversity suggests broad functional redundancy in these soil communities within sites, where taxa may shift with succession as the environment and resource availability change but broad function remains constant (45). However, key groups of microbes that have important interactions with plants showed clear differences between our two sites and the successional stages within them, some of which mirrored aboveground changes in plant community composition and diversity and others that did not. Our results also demonstrate that predictions generated for soil microbes in moist tropical forests may not apply to dry forests.

Our finding that the diversity of the total bacterial and fungal communities does not change as forest succession proceeds and plant diversity increases is in contrast with our predictions. Relationships between plant and microbial diversity can be highly variable, with some studies showing that microbial richness increases as sites recover from disturbances and forest cover establishes (37, 46) and others showing no variation or decreasing levels of richness as forests age and plant diversity increases (1, 13, 16). Our sampling was replicated and sequencing was “deep” (Fig .3) such that any potential differences in diversity should have been identified. Furthermore, our study sites were carefully chosen to have similar underlying geology and soils across successional stages to minimize the effects of soil chemistry in driving microbial community shifts.

Differences in landscape-scale forest recovery at our two sites are likely influence some of the community patterns that we see. Much of the Los Santos region of Panama was dry tropical forest before it was cleared during the 1950s but the land is now predominantly maintained as cattle pasture with forest patches being very isolated (47, 48). Nevertheless, the largest expanse of mature dry forest in Panama, at Achotines Laboratory, is adjacent to our study site, providing propagule sources for both plants and microbes. Agua Salud, our moist tropical forest site adjacent to the former Canal Zone of Panama, also experienced deforestation in the early-mid 20th century, but today there is less agriculture and more secondary forests have developed as pastures have transitioned from fallows to forests. Soberania National Park, a 220 km^2^ area of mature forest, is also adjacent. Thus, our sites have similar land use histories, but today the landscape matrix differs and may be influencing the dispersal of microbial taxa between pastures and the forests that surround them and driving the larger community differences between successional stages that are seen at the dry site for both bacteria and fungi.

There is a growing body of work highlighting feedback between plants and microbes, emphasizing that both mutualists and pathogens are more likely to be generalists with multiple hosts rather than plant species-specific (33, 38, 49, 50). We found that the diversity and community structure of important functional groups of microbes to be strikingly different between sites (Fig. 3a through c) and, in some cases, successional stage, suggesting that both plant hosts and abiotic parameters may be affecting their distribution at the greater landscape level. For one, pasture grasses may be expected to associate with different microbes than woody plants (51). Previous work has shown that most neotropical trees associate with AMF but the strength of their interactions can vary depending on successional stage and soil fertility, with species that have obligate associations with AMF more likely to be dominant in low nutrient soils and late successional forests (52). The diversity of fungal pathogens is strongly influenced by humidity and temperature (53), and as plant diversity increases with succession, pathogen diversity typically increases as more hosts become available ([54]) but see reference [13).

Both sites had clear differences in the communities of AMF between pastures and forested areas, as seen in other studies in both temperate and tropical environments (5, 55–58). AMF can persist for decades in long-term pastures and may facilitate the establishment of forests when agricultural land is abandoned (3). However, these AMF communities showed little overlap at our dry and moist sites. Previous work in tropical regions has suggested that increased plant diversity should lead to a higher diversity of AMF, due to host-AMF specificity (20) in which case our moist site should have had higher AMF diversity. We observed this pattern within sites, with Pastures having the lowest diversity and OSF the highest (but significant differences only at the moist site). However, the dry site showed higher relative abundance, higher overall diversity, and higher community turnover of AMF than the moist site, with each successional stage having distinct communities and many more ASVs showing significant differences between stages (55 dry ASVs vs 16 moist ASVs, LDA = 4.0, *P* < 0.01), despite the diversity of tree species being lower (Fig. 1a and 3a ). AMF generally have large spores and can survive harsh conditions (56). In these dry forests, host plants may depend on and associate with a more diverse community of AMF to survive the variable conditions present during the longer dry season. It is also possible that at our moist site where soil phosphorus is more limiting, plants interact with a subset of AMF that are particularly good at solubilizing soil phosphorus, causing these taxa to dominate the AMF community (59). Thus, AMF diversity is high across the greater landscape but community composition may be driven by site conditions, such as soil moisture and nutrient availability which can vary across succession, in addition to the selectivity of their plant hosts.

Biological nitrogen fixation, which converts atmospheric N_2_ into ammonia making it accessible to plants, is one of the most important benefits that plants gain from interacting with bacteria. Previous work has shown that the importance of associations with N_2_-fixing bacteria can vary with successional stage, with leguminous N_2_-fixing trees in younger forests having higher rates of nodulation and fixation than N_2_-fixing trees in older forests (29). In our study, the proportion of legume trees in YSF was three times higher than OSF at both of our sites yet the diversity and overall relative abundance of N_2_-fixing bacteria in the bulk soil was similar in the two successional stages at both sites. This confirms that the capacity for symbiotic N_2_ fixation persists in these forests throughout succession, even if the proportion of trees that are fixing nitrogen declines over forest age (29). We also note that the communities of putative N_2_-fixing bacteria showed more overlaps between sites and successional stages than the total bacterial community, largely because of the high relative abundance of shared *Bradyrhizobium* ASVs across all samples at both sites. This genus is the most common symbiont of legume root nodules in the moist forests of central Panama (60, 61) and probably also in these dry forests, clearly making it a generalist symbiont as only 20% of legume tree species were shared between our two study sites. However, Pastures also showed a high relative abundance of *Bradyrhizobium* in their soil communities and as there are members of the *Bradyrhizobium* that are incapable of nodulating legume roots and cannot fix nitrogen (62), we cannot exclude the possibility that free-living *Bradyrhizobium* is ubiquitous in these soils.

Overall, our dry site had lower diversity and abundance of leguminous trees but a higher diversity of potential symbionts than the moist site, suggesting that the plants at our dry site might associate with a more diverse community of N_2_-fixing bacteria, although work looking directly at plant nodule communities is needed to confirm this. As seen with the AMF community, the higher diversity of N_2_-fixing bacteria in the dry site may relate to the tolerance of their hosts to the seasonal availability of water. Most trees in our dry forest are deciduous and must rebuild their leaf area annually when the rainy season begins. Legumes may associate with a more diverse community of N_2_-fixing bacteria to maximize nitrogen acquisition by avoiding downregulation of fixation at certain times of the year when drought stress is high and use the acquired nitrogen to synthesize photosynthetic enzymes, allowing internal CO_2_ levels in the leaves to drop and photosynthesis to occur with lower stomatal conductance and reduced transpiration (63). It is also possible that the higher pH of the soils at our dry site allows for a more diverse community of N_2_-fixing taxa to persist (38).

The quantity and quality of soil resources have been hypothesized to influence the selection of pathogenic organisms. We found that pathogen richness and communities differed by site and successional stages, with higher diversity and relative abundance at our moist site, particularly in OSF (Fig. 3c; Table S3b). Only eight genera of putative pathogens were observed in forests at the dry site, and only *Ceratobasidium* was identified across all successional stages at both sites. This is consistent with previous work suggesting a higher diversity of pathogens in Neotropical forests with lower light and higher moisture (33, 49, 64). The lack of overlap in the pathogen community between the moist and dry forests is similar to the differences in the aboveground tree communities, suggesting a distribution for these pathogens which may be linked to available host plants. However, the abiotic environment is clearly influential and, while we cannot disentangle the potential influences of higher precipitation/lower soil pH and nutrients with lower precipitation/higher soil pH and nutrients, available evidence suggests that moisture may be the more important driver of pathogen abundance and diversity in these forests. Spear et al. (64) compared pathogen pressure between forests in the former Canal Zone of Panama with rainfalls of 1,800 and >3,000 mm/year and found that pathogens likely play a role in excluding seedlings of dry forest species from wetter forests where pathogen pressures are higher as they are more likely to die from pathogen attack. The higher proportion of deciduous trees in the dry forest may also reduce pathogen pressure by depriving pathogens of suitable hosts for several months of the year (65). We acknowledge that disease development is context dependent and that many fungal pathogens can transition between endophytic, saprotrophic, and pathogenic lifestyles so there is some level of uncertainty when broadly categorizing soil taxa as pathogens. While all of the genera that we highlight here have broad global distributions, on a local scale we see major shifts in community between our two study sites. However, given that the majority of work studying plant-pathogen interactions in the region has been in the lowland wet and moist forests surrounding the Panama Canal (reviewed in reference (33)), our inability to identify many potential pathogens suggests a need for more plant pathology work in soils and plants of neotropical grasslands and dry tropical forests.

We also note that differences were found in the distribution of microbes with the potential to inhibit plant pathogens. Endophytic fungi, including AMF, have been shown to inhibit pathogen damage on plants (66, 67) and AMF are more diverse and abundant at our dry site. By contrast, *Trichoderma* (Ascomycota)*,* which can be pathogenic in some contexts but also has anti-fungal properties, was only found at our moist site where it had a relative abundance that was an order of magnitude higher in forested plots than in Pastures (Fig. 3c, Table S3b). At our dry site, the higher diversity and relative abundance of Actinobacteria and their associated antimicrobial activity could contribute to the reduced incidence of plant pathogens. For example, the genus *Streptomyces* (Actinobacteria) was two to three times more common in dry than moist forest plots and was found only in OSF plots at the moist site. Members of the *Streptomyces* have been shown to produce many secondary metabolites and can suppress plant pathogens in the dry forests of Costa Rica (68). Soil-borne pathogens exist in complex microbial communities and interactions with other soil microbes may also play a role in their distribution, as well as their pathogenic impacts. While we cannot demonstrate direct interactions between microbes or plants with our data, the broad scale differences that we document between sites and successional stages suggest many avenues of research that are needed to better understand them.

Our finding of higher diversity and unique communities of key microbial taxa, such as AMF and N_2_-fixers in the dry forests of Los Santos emphasizes the importance of tropical dry forests, a globally threatened habitat, as reservoirs and hot spots of microbial diversity. Our results support the hypothesis that mutualisms that increase nutrient and water uptake, such as AMF and N_2_-fixers, are more important for forest communities in environments with low water availability (23). If there is a stronger host-microbe interdependence in drier habitats, progressive habitat loss and fragmentation of these landscapes will be more likely to result in a situation where human-induced dispersal barriers inhibit the arrival of key taxa that are necessary for ecosystem functioning and service provision in the forests that remain (41). Indeed, the much higher overall levels of dissimilarity among successional stages, for nearly all microbial phyla, in our dry compared to our moist site does suggest that the much lower forest cover and higher fragmentation at the dry site limits microbial dispersal across the landscape.

Our results are encouraging from the standpoint of restoration and forest conservation as they point to rapid changes in soil microbial communities following the abandonment of cattle pastures and the natural regrowth of forests in the lowland tropics. Secondary tropical forests are increasingly recognized as being important for their ecosystem services in these patchy landscapes, such as accumulating carbon (69), reducing soil respiration (70), enhancing water quality (71), improving seasonal stream flow (72), and conserving biological diversity (73). Mixed landscapes have also been shown to preserve belowground biodiversity—particularly in dry sites (74). However, reduced forest connectivity and patchiness can pose challenges for passive restoration through natural forest recovery due to the dispersal limitation of not only trees (75–77) but also soil microbes. Active reforestation, which includes planting small patches of native trees as well as maintaining forested riparian corridors across the landscape, may enhance belowground microbial communities as well as provide a variety of other biodiversity and ecosystem benefits in mixed landscapes undergoing succession (77).

While much is yet to be learned about the role of specific microbes in cycling nutrients and providing other ecosystem services, soil communities, including microbes that have strong interactions with forest plants, in these young forests showed strong signs of assembly to communities that were similar to older forests after only two decades of regrowth. Given the pronounced differences in microbial communities between our dry and moist forest sites, our study also points to the importance of considering active management of the forest microbiome, such as inoculation with biofertilizers composed of locally adapted microbial ecotypes, as climate-resilient forests are planted to secure the above-mentioned ecosystem services.

## MATERIALS AND METHODS

### Site descriptions

Our study was conducted at two sites that represent moist and dry tropical lowland forests in Panama. The two sites have distinct tree species composition and richness, with relatively little species overlap.

The moist forest site was located in the Agua Salud Project experimental site in the northern Panama Canal Watershed (9°12′ 38″N; 79°44″ 35″W). The mean annual rainfall at Agua Salud is 2,700 mm, with the dry season from December to April. Soils are strongly weathered, infertile, and well‐drained Oxisols and Inceptisols (2) with a high clay content and a mean pH of around 5.0 (43). The area is a matrix of secondary forests of multiple ages, actively grazed cattle pastures, and small areas managed as subsistence farms. Today, much of the surrounding area is undergoing natural succession (69).

Our dry forest site was located in the province of Los Santos, at the southern tip of the Azuero Peninsula (7°25′30″N; 80°10′30″W). The region experiences mean annual rainfall of 1,680 mm and typically has 5–6 months of dry season (December–May). Soils in Los Santos are rocky, with basalt as the underlying geology, and have textures ranging from clay loam to clay (78) and a mean pH of around 6.0. Much of the region was cleared by the 1950s for cattle ranching (48). Today, the area is dominated by active pastures planted with exotic grasses (primarily *Hyparrhenia rufa* (Nees) Stapf and *Megathyrsus maximus* Jacq.), isolated trees, and living fences, with remnant forest patches mostly in riparian areas and abandoned pastures with naturally regrowing secondary forest being rare (78).

These two sites were chosen because they both contain young (15–20 y) and old (>80 y) secondary forests (hereafter, YSF and OSF) that were previously managed as cattle pastures for at least 20 years and have naturally regenerated following pasture abandonment. Pastures at both sites have been under low-intensity management, with some use of fire but no-tillage or use of herbicides or fertilizers, and were actively grazed at the time of sampling. Climatic parameters, such as rainfall and mean air temperature, are consistent within each site. Most importantly, extensive sampling has shown soils to be remarkably homogeneous within sites (43, 44, 79 /this study) allowing us to make comparisons between successional stages without the confounding factors of variation in soil pH and nutrients (Table S1).

Secondary forest monitoring networks were established at both sites in 2008 and used the same sampling methods. A complete description of plot size and network layout, as well as a detailed analysis of secondary forest dynamics and diversity at each site, can be found in van Breugel et al. (43) and Estrada-Villegas et al. (44). At our moist site, we selected plots in the YSF and OSF that are physically located hundreds of meters apart and our pastures were interspersed in the YSF across the site. At the dry site, YSF and OSF sampling plots were more clustered due to the distribution of forest patches in the area while pastures were separated by larger distances. Plot layout in pastures was as in the secondary forest where temporary 20 × 50 m plots were established for soil sampling. Pasture vegetation was not inventoried but dominant grasses are noted above; these plots did not contain any trees. Here we present the first between-site comparison of tree species richness and species composition (stems with a DBH ≥5 cm). Differences in species composition among plots were calculated as the Ružička dissimilarity index and visualized using non-metric multidimensional scaling (NMDS), using the R package *vegan* (79).

### Soil sampling and DNA extraction

We collected 36 surface soil samples (0–15 cm depth) at each site during the wet season. Four permanent plots within each successional stage were sampled, with three cores sampled along a single transect in each plot and 20 m spacing between cores (12 samples per successional stage per site). These existing plots are used for long-term vegetation monitoring in our moist (43) and dry forest sites (44). Cores were placed in Ziploc bags immediately after collection and kept on ice in a cooler on the day of collection until arriving at the laboratory where samples were thoroughly mixed and subsampled for nutrient and molecular analysis. Individual subsamples for microbial analysis were frozen at −20 °C until DNA was extracted from 25 mg of soil with MOBIO PowerSoil DNA Extraction kits using the manufacturer-recommended protocol.

### Soil nutrient analyses

Soil nutrients were analyzed using procedures detailed in Turner and Romero (80). Briefly, soil pH was determined in a 1:2 soil/water ratio with 0.01 M CaCl2. Extractable Al, base cations (Ca, K, and Mg), P, and micronutrients (Cu, Fe, Mn, and Zn) were determined by extraction in Mehlich-III solution and detected with inductively coupled plasma optical-emission spectrometry (ICP-OES) on an Optima 7300 DV (Perkin Elmer, Inc., Shelton, CT, USA).

### Metabarcoding library preparation and sequencing

Microbial communities were assessed using high-throughput sequencing to characterize their distribution and abundance. Bacteria were amplified by targeting the V4 hypervariable region of the 16S rRNA gene using the 515F–806R primer pair (81). For fungi, we amplified the first internal transcribed spacer (ITS1) region of the rRNA operon, using the primers ITS1F (82) and ITS2 (83). Primers included all necessary Illumina adapters with barcodes to distinguish samples. The bacterial 806R primer included a 10 bp barcode sequence while the ITS1F and ITS2R primers each included an 8 bp barcode sequence, allowing us to multiplex multiple samples. We used 5Prime Hot MasterMix (QuantaBio) in PCRs with a final volume of 12.5 μl with 25 cycles using a 50 °C annealing temperature for both loci. PCRs were done in triplicate, pooled, and products were cleaned and normalized using Sequelprep Normalization plates (Thermo Fisher). We also amplified a portion of the 18S rRNA region, using primers AMV4.5NF and AMDGR (84), to further examine AMF communities. AMF amplifications were done using 2-stage PCR with Platinum Hot Start PCR Mastermix (Thermo Fisher) where the locus-specific primers used for PCR1 included the Illumina sequencing primer sequence on their 5′ ends. This first PCR included 30 cycles with an annealing temperature of 50 °C. PCR2, done with 1 μl of PCR1 product and six amplification cycles, added on remaining Illumina adapters as well as 8 bp barcodes on either end of the amplification products. AMF amplicons were cleaned and normalized using PCR Normalization plates (Charm Biotech). For all loci, cleaned amplification products were pooled together by locus, and the three libraries were concentrated using AMPure beads. Libraries were quality checked on a BioAnalyzer (Agilent), concentrations were estimated using a Qubit fluorometer (Thermo Fisher), and sequenced on separate MiSeq runs with 2 × 250 bp paired-end reads. All sequencing was conducted at the Naos Molecular Laboratory (STRI).

### Bioinformatics

Raw sequence reads were demultiplexed, trimmed to remove adapter and primer sequencers, and filtered and corrected using *DADA2* (85) and associated packages such as *cutadapt* (86) implemented in R (v. 3.6.1) prior to constructing amplicon sequence variant (ASV) tables. Taxonomy was assigned using SILVA databases for the 16S (v.138;^87^ ) and AMF (v. 132, 88) data sets and UNITE (February 2020 release; 89) for the ITS data set. For fungi, we used the “dynamic” similarity threshold. All ASVs that were not assigned to a Kingdom or to a non-microbial taxonomic group (e.g., Eukaryota in the 16S data set, Amoebozoa or Arthropoda in the AMF data set) were removed prior to downstream analysis. This filtering step reduced the total ASV count from 26,677 to 26,266 for 16S, and from 4706 to 1490 ASVS for AMF (Only ASVs assigned to the class “Glomeromycetes” were retained). All ITS ASVs (10,644) were assigned to the Kingdom Fungi and were retained. Sequencing depths were adequate to capture the diversity within all microbial communities (Fig S3) and data were rarefied prior to alpha diversity analyses to a level of 18,000 sequences/sample for bacteria (24,267 ASVs remaining), 17,000 sequences/sample for fungi (9,983 ASVs remaining), and 6000 sequences/sample for AMF (1486 ASVs remaining). One Pasture sample from our moist site was eliminated from the analysis due to poor amplification and low sequence coverage.

To further explore guilds of microbes that associate with plants beyond AMF, we categorized subsets of sequences identified to the genus level into different functional groups that are known to have ecologically important interactions with plants. We acknowledge that identification of any genus with metabarcoding is crude as these sequence fragments are short and may have limited taxonomic resolution, most genera include many different species that may have different functional significance, and their function may be context specific. However, this approach allows us to look at trends worthy of further exploration.

Arbuscular mycorrhizal fungi included all ASVs assigned to the phylum Glomeromycota when sequenced with ITS primers, or the Class Glomeromycetes, when sequenced with AMF-specific 18S primers. Nitrogen (N_2_)-fixing bacteria included both free-living and plant-associated taxa that have been shown to have the ability to fix nitrogen (Table S3a; Fig. S1). The majority of these are members of the phyla Proteobacteria or Actinobacteriota. Our potential fungal pathogen data set included the subset of genera that are classified as “highly probable” or “probable” plant pathogens by FUNGuild (90) and that have previously been identified in Panama through isolations from diseased tissues (Table S3b; 33, 64, 91). All of these taxa are members of the Ascomycota.

We compared the diversity of communities with alpha and beta diversity statistics and ordination techniques on the rarefied data sets using the R packages *phyloseq* (92), *vegan* (93)*,* and *microbiome* (94). Shannon and Inverse Simpson metrics (results not shown) were calculated as measures of alpha diversity and Kruskal-Wallis tests, followed by paired Wilcox tests with fdr correction, were used to look at differences between sites and successional stages. We looked at differences in community composition of the total bacterial and fungal and AMF communities by calculating Bray-Curtis distances using rarefied data sets; analyses of N_2_-fixing bacteria and plant fungal pathogens were done using sequences subset from the rarefied data sets. Data were Hellinger transformed to minimize the impact of outlier samples and patterns of beta diversity were visualized with Principle Coordinates Analysis plots. To test for the effects of successional stage and soil chemistry on microbial communities, we used the *vegan* package (v2.5–7) to first calculate the beta dispersion of the distance matrices (betadisper function), then performed a Permutation Test for Homogeneity of multivariate dispersions (permutest function), and finally PERMANOVA (adonis2 function with 999 permutations; assuming equal dispersion) or Analysis of Similarity (ANOSIM; where beta dispersion was significant).

Comparisons of taxonomic shifts between sites and/or successional stages were done on normalized data with Kruskal-Wallis tests followed by post hoc FDR correction. Linear discriminant analysis Effect Size (LEfSe), as implemented on the Microbiome Analyst website (95), was used to test for differential abundance of taxa between successional stages within each site using an LDA threshold of 4.0 and adjusted *P* cut-off of 0.05.

## Data Availability

All supporting data files, including sample metadata, R scripts, and ASV abundance tables can be accessed through Smithsonian Figshare, at https://doi.org/10.25573/data.27934533.v1. Raw fastq files and metadata can be found in the NCBI SRA archive in BioProject PRJNA1159263.

## SUPPLEMENTAL MATERIAL

Supplemental figures and tables spectrum.01931-24-s0001.pdf Fig. S1 to S3, Tables S1 to S4, including rarefaction curves, functional groupings of N fixing bacteria and putative plant fungal pathogens, phylum level results, and statistical analyses.

ASM does not own the copyrights to Supplemental Material that may be linked to, or accessed through, an article. The authors have granted ASM a non-exclusive, world-wide license to publish the Supplemental Material files. Please contact the corresponding author directly for reuse.
