# Supplementary material for: Soil microbial communities in dry and moist tropical forests exhibit distinct shifts in community composition but not diversity with succession
Source: Microbiol Spectr. 2025 Feb 4;13(3):e01931-24. doi: 10.1128/spectrum.01931-24 (PMC11878062; doi:10.1128/spectrum.01931-24)
Supplement: Supplemental figures and tables — Fig. S1 to S3, Tables S1 to S4, including rarefaction curves, functional groupings of N fixing bacteria and putative plant fungal pathogens, phylum level results, and statistical analyses. [file spectrum.01931-24-s0001.pdf]

Soil microbial communities in dry and moist tropical forests exhibit distinct shifts in community composition but not diversity with succession

Supplemental Material

Kristin Saltonstall*, Michiel van Breugel, Wayra Navia, Hilda Castillo, Jefferson S. Hall

**Corresponding authorl:* saltonstallk@si.edu

Figure S1 | Responses of dominant bacterial Phyla to succession at moist and dry forest sites in Panama. Successional differences in Shannon diversity estimates were calculated from rarefied data and compared within sites using Kruskal-Wallis tests followed by Wilcox tests with FDR adjustment. Letters indicate significant differences between successional stages within a site ($p<0.05$). Compositional similarity of microbial communities (beta diversity) is represented using Principal Coordinates Analysis (PCoA) ordination plots of Bray-Curtis distance matrices calculated from rarefied data that was Hellinger transformed. Numbers in brackets indicate the percent of the total variance explained by each axis. Histograms of relative abundances were plotted from data that was normalized using total sum scaling. P = Pasture, YSF = Young Secondary Forest, OSF= Old Secondary Forest. PERMANOVA analysis showed Pasture communities to be different from YSF and OSF communities at the moist site for all phyla (pairwise adonis $p<0.05$) whereas all three successional stages had different communities for all phyla at the dry site ($p<0.05$).

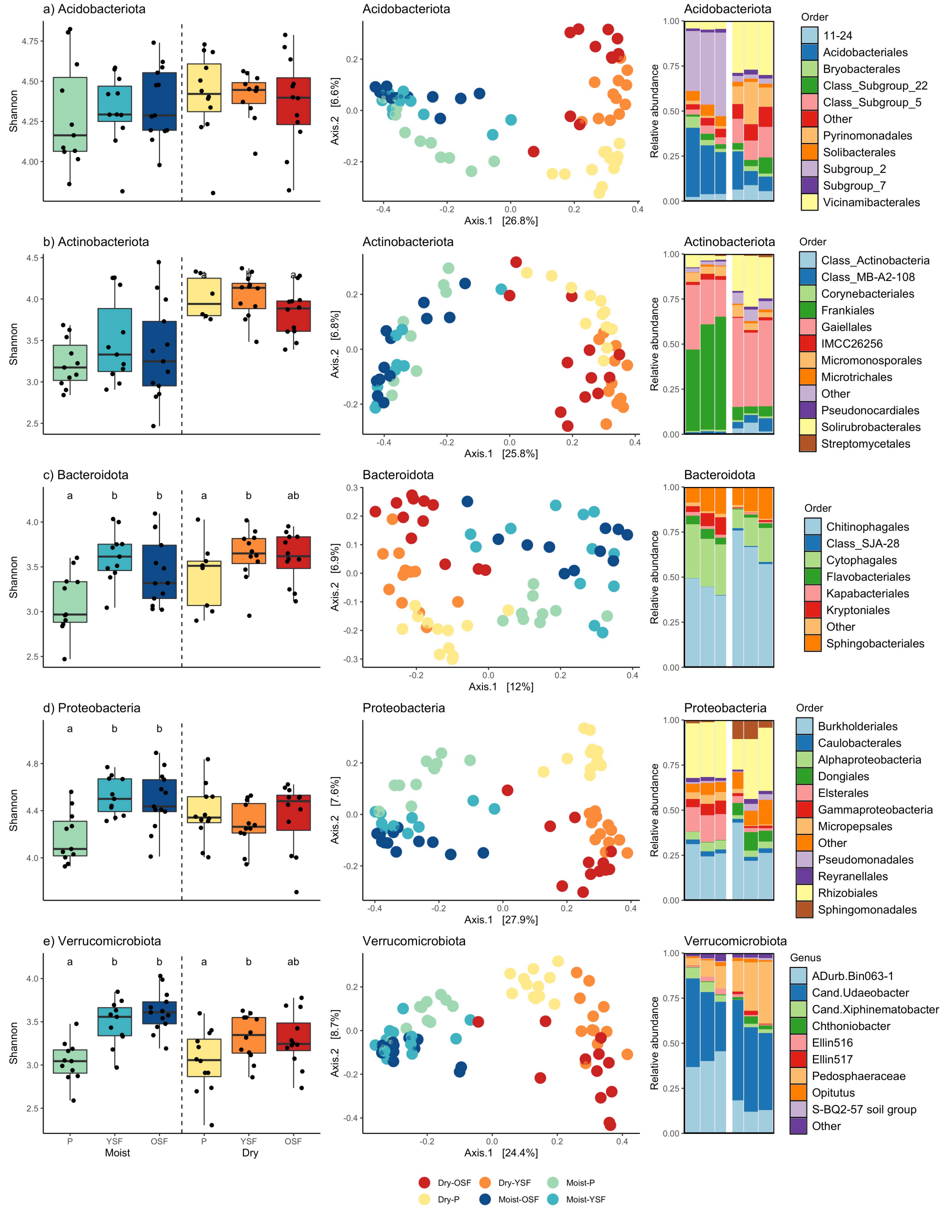

Figure S2 | Responses of dominant fungal Phyla to succession at a moist and dry forest site in Panama. Successional differences in Shannon diversity estimates (Alpha Diversity) were calculated from rarefied data and compared within sites using Kruskal-Wallis tests followed by Wilcox tests with FDR adjustment. Letters indicate significant differences in diversity between successional stages within a site ($p<0.05$). Compositional similarity of microbial communities (Beta diversity) is represented using Principal Coordinates Analysis (PCoA) ordination plots of Bray-Curtis distance matrices calculated from abundance filtered data that was Hellinger transformed and normalized using total sum scaling. Numbers in brackets indicate the percent of the total variance explained by each axis. Histograms of relative abundances were plotted from data that was normalized using total sum scaling. P = Pasture, YSF = Young Secondary Forest, OSF= Old Secondary Forest. PERMANOVA analysis showed communities of Basidiomycota and Glomeromycota in Pastures to be different from YSF and OSF communities at the moist site (pairwise adonis, $p<0.05$) whereas all three successional stages had different communities for all phyla at the dry site ($p<0.05$).

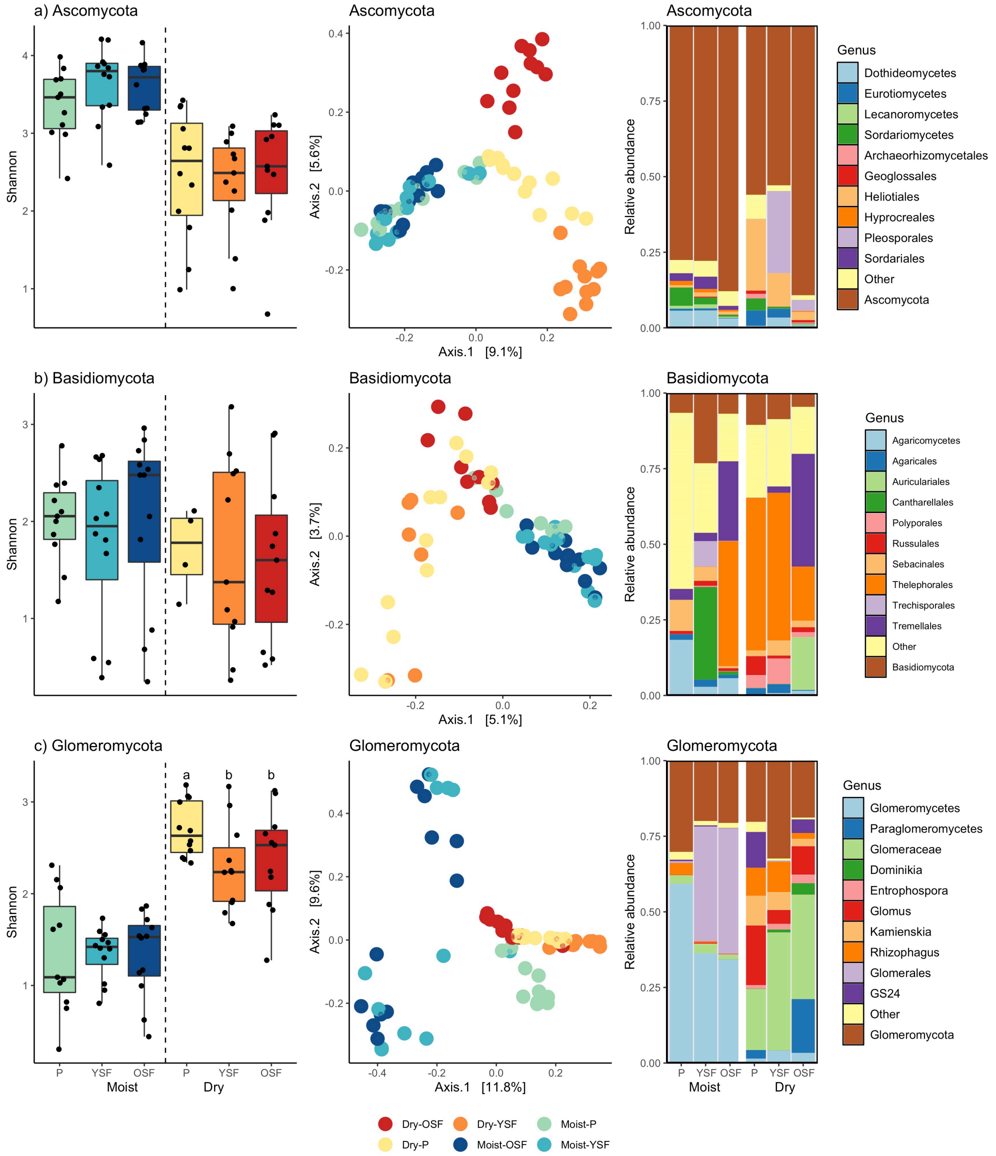

Figure S3: Rarefaction curves of a) bacterial, b) fungal, and c) AMF sequences from soil samples collected at moist (n=35) and dry (n=36) tropical forests in Panama.

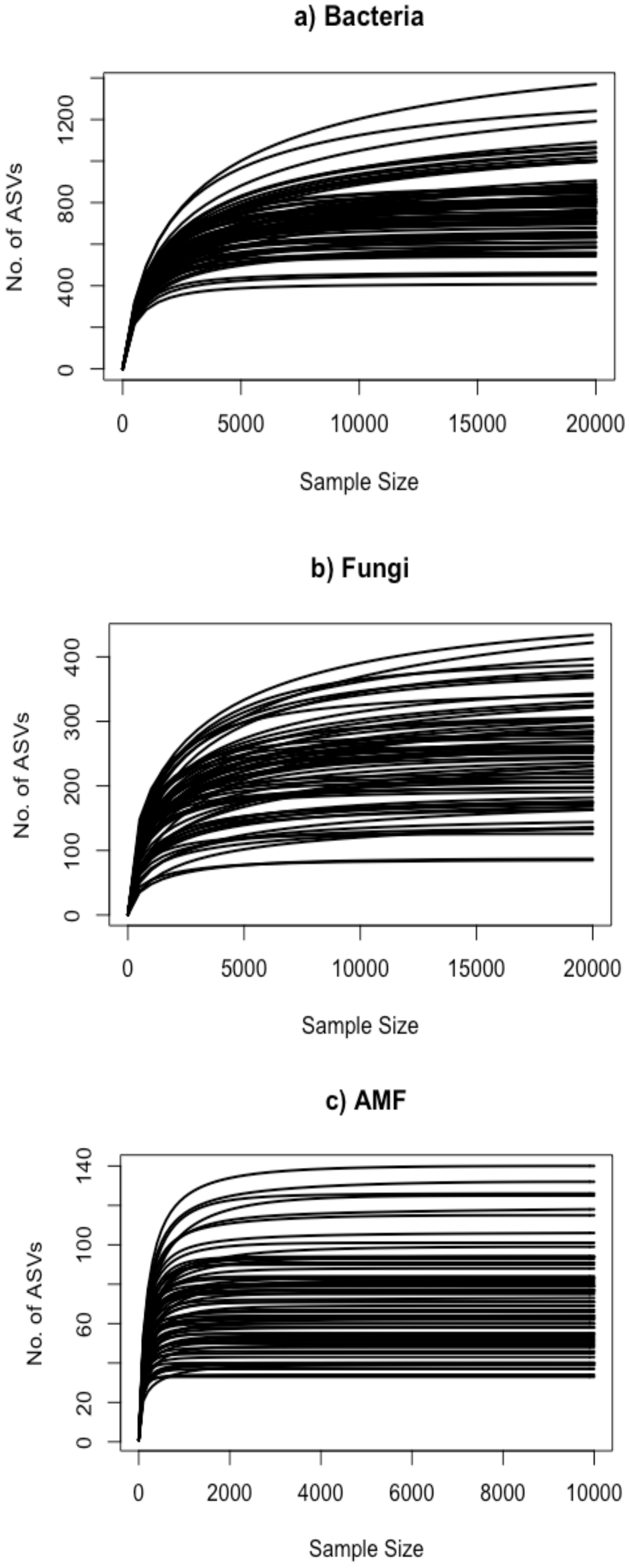

Table S1: **Soil chemistry across successional stages at a moist and a dry site in Panama**. All measurements are means ± SD of 12 cores (0-15 cm depth) collected in each successional stage by site. Soil properties highlighted in grey have significant differences between sites for soil chemistry properties (Kruskal-Wallis test, $p<0.05$). Letters in superscript refer to significant differences ($p<0.05$) among successional stages within a site based on pairwise Wilcox tests with post-hoc Bonferroni correction ($p<0.05$). YSF = Young secondary forest, OSF = Old secondary forest

	Moist Forest			Dry Forest		
	**Pasture**	**YSF**	**OSF**	**Pasture**	**YSF**	**OSF**
Soil property						
pH (H2O)	5.1 ± 0.1	5.1 ± 0.3	5.0 ± 0.3	5.9 ± 0.2	5.9 ± 0.2	6.0 ± 0.5
pH ($CaCl_2$)	4.8 ± 0.2	4.7 ± 0.4	4.8 ± 0.3	5.7 ± 0.2	5.6 ± 0.5	5.7 ± 0.5
Al (mg/kg)	941.4 ± 107.4[a]	1036.5 ± 104.8[b]	951.8 ± 119.8[a]	860.7 ± 88.7[a]	935.0 ± 53.9[a]	750.2 ± 132.6[b]
Ca (mg/kg)	771.6 ± 568.1	755.9 ± 576.6	912.9 ± 290.9	5039.4 ± 588.7	5411.0 ± 382.8	4572.5 ± 2105.1
Cu (mg/kg)	5.7 ± 1.9[a]	3.8 ± 1.1[b]	3.7 1.2[b]	8.3 ± 2.9[a]	4.2 ± 0.9[b]	4.1 ± 2.3[b]
Fe (mg/kg)	199.2 ± 45.4	213.6 ± 29.6	192.6 ± 43.5	242.0 ± 52.6[a]	183.0 ± 16.2[b]	163.2 ± 44.3[c]
K (mg/kg)	97.2 ± 70.5[a]	45.6 ± 22.1[b]	51.6 ± 23.6[b]	158.6 ± 156.2[a]	45.5 ± 24.0[b]	95.1 ± 84.9[a]
Mg (mg/kg)	427.7 ± 343.2	399.5 ± 393.3	448.6 ± 129.2	1476.4 ± 289.6[a]	1479.5 ± 192.0[a]	1030.1 ± 363.6[b]
Mn (mg/kg)	208.3 ± 73.8[a]	369.5 ± 95.1[b]	236.5 ± 130.2[a]	127.2 ± 31.7[a]	85.6 ± 43.9[b]	145.9 ± 62.8[a]
P (mg/kg)	2.4 ± 0.7	2.5 ± 0.6	2.3 ± 0.8	4.7 ± 3.7[a]	2.9 ± 1.0[a]	8.0 ± 4.4[b]
Zn (mg/kg)	2.9 ± 1.8[a]	3.2 ± 0.7[a]	1.8 ± 1.9[b]	5.3 ± 1.7	4.8 ± 3.1	6.7 ± 12.7

Table S2: **Soil microbial community composition changes with succession**. Total numbers of ASVs and mean relative abundance of dominant taxa (>1% average within a successional stage) of a) Bacteria, b) Fungi, and c) Arbuscular Mycorrhizal Fungi (AMF) at Moist and Dry sites in Panama. Groups highlighted in grey showed significant differences in abundances between the Moist and Dry forest sites based on LefSe analysis (LDA=4.0, p<0.05 with FDR correction). Letters in superscript indicate significant differences in relative abundance between successional stages within sites (LDA=4, p<0.05 with FDR correction). ASVs = number of ASVS identified within the taxonomic group at each site, YSF = Young secondary forest, OSF = Old secondary forest. "Other" includes phyla with a relative abundance of <1.0% and Unidentified Bacteria or Fungi

a) Bacteria (16S)

	Moist Forest				Dry Forest			
**Phylum**	**ASVs**	**Pasture**	**YSF**	**OSF**	**ASVs**	**Pasture**	**YSF**	**OSF**
Acidobacteriota	2217	30.3	28.6	30.0	1521	22.5[a]	19.1[b]	22.9[a]
Actinobacteriota	654	3.8	2.8	3.3	860	12.4[a]	19.1[b]	11.7[a]
Bacteroidota	918	2.6[a]	3.9[b]	2.7[a]	924	4.8[a]	7.3[b]	7.8[b]
Chloroflexi	1894	14.7[a]	8.3[b]	8.2[b]	965	4.1[a]	7.9[b]	3.6[a]
Firmicutes	205	0.4	0.1	0.2	119	2.3	1.0	2.7
Gemmatimonadetes	187	1.3[a]	1.2[a]	2.4[b]	280	3.3	2.7	2.8
Methylomirabilota	79	0.9	1.5	2.1	42	1.7[b]	1.4[a]	2.1[c]
Myxococcota	1034	8.9	9.4	10.3	659	10.2[a]	3.3[b]	4.6[b]
Nitrospirota	42	<0.1[a]	0.2[a]	1.0[b]	24	0.9[a]	0.1[b]	0.8[a]
Planctomycetota	1653	2.8	3.9	2.9	1027	2.3[a]	4.1[b]	4.3[b]
Proteobacteria	2606	16.6[a]	21.2[b]	21.1[b]	1505	21.0	17.6	18.2
αProteobacteria	1461	10.1[a]	13.5[b]	13.0[b]	778	10.3	11.7	10.0
γProteobacteria	1093	8.1	6.5	7.7	708	10.7[a]	5.9[b]	8.2[c]
RCP2-54	64	0.9	1.1	1.2	27	0.9[a]	0.4[b]	1.0[a]
Verrucomicrobiota	1176	13.2 [a]	12.3[a]	8.8[b]	586	8.8[a]	12.1[b]	11.4[b]
Other Bacteria	3494	4.6	6.6	7.1	1362	5.7	4.4	7.1

b) Fungi (ITS1)

	Moist Forest				Dry Forest			
**Phylum**	**ASVs**	**Pasture**	**YSF**	**OSF**	**ASVs**	**Pasture**	**YSF**	**OSF**
Ascomycota	2773	67.8	65.0	64.9	1101	39.0[a]	70.7[b]	62.0[b]
Basidiomycota	815	3.9[a]	12.2[b]	20.1[c]	562	21.2	11.7	18.8
Glomeromycota	140	1.8	2.4	1.6	442	12.5[a]	6.0[b]	2.4[b]
Other Fungi	2478	26.6	20.4	13.4	1894	27.3	11.7	16.8

c) AMF (18S)

	Moist Forest				Dry Forest			
Taxonomic Group	**ASVs**	**Pasture**	**YSF**	**OSF**	**ASVs**	**Pasture**	**YSF**	**OSF**
Glomeraceae								
Glomus	52	7.6	6.3	6.7	25	1.6[a]	2.8[a]	10.4[b]
Rhizophagus	94	34.6	34.1	25.8	93	15.9	15.1	13.4
Septoglomus	0	NA	NA	NA	49	17.4[a]	1.8[b]	4.7[b]
Unidentified	29	2.7[a]	4.3[b]	6.0[b]	21	1.7	2.6	2.3
Gigasporaceae								
Gigaspora	16	<0.1	0.2	0.3	4	0.2	0.0	<0.1
Racocetra	3	0.0	<0.1	<0.1	3	<0.1	0.0	<0.1
Unidentified	1	0.0	<0.1	0.0	1	0.0	0.0	<0.1
Acaulospora	35	0.6	0.5	0.4	42	2.5	<0.1	0.7
Ambispora	5	0.2	0.0	0.0	0	NA	NA	NA
Claroideoglomus	3	0.3	0.0	0.0	24	1.8	0.6	<0.1
Paraglomus	1	<0.1	0.0	0.0	25	2.6	<0.1	1.9
Sacculospora	0	NA	NA	NA	3	<0.1	0.0	<0.1
Other AMF								
Order_Diversisporales	0	NA	NA	NA	6	0.2	<0.1	0.0
Order_Glomerales	308	43.7	46.0	53.3	473	42.1	64.7	53.0
Class_Glomeromycetes	99	10.2	8.5	7.5	189	14.0	12.3	13.4

Table S3: **Functional groups of microbes associated with plants**. A) Genera of putative Nitrogen-fixing bacteria included in the Nitrogen-Fixer Functional Group. Columns 3-8 indicate the percent relative abundance of each Genus by forest type and successional stage within the N_2-fixing community which was subset from the total rarefied bacteria dataset. B) Genera of putative plant fungal pathogens included in the Plant Pathogen Functional Group, subset from the total rarefied fungal dataset; all are known plant fungal pathogens that have previously been isolated from diseased leaf tissues in Panama. Columns 2-7 provide the percent relative abundance of each genus by forest type and successional stage within the plant fungal pathogen communities. Letters in superscript indicate significant differences in relative abundance between successional stages within sites (LefSe, LDA=4, $p<0.05$ with FDR correction). YSF = Young Secondary Forest, OSF = Old Secondary Forest.

a)

		**Moist Forest**			**Dry Forest**		
**Genus**	**Habitat**	**Pasture**	**YSF**	**OSF**	**Pasture**	**YSF**	**OSF**
Azoarcus	Free living	0.0	0.0	0.0	3.3[a]	0.0[b]	0.0[b]
Azospirillum	Free living	0.0	0.0	0.0	0.1	0.1	0.0
Bacillus	Free living	6.5	2.8	5.0	38.0[a]	2.8[b]	4.4[b]
Beijerinckia	Free living	0.6	<0.1	0.1	0.0	0.0	0.0
Microvirga	Free-living	0.0	0.0	0.0	4.2	3.4	3.3
Paenibacillus	Free living	0.2	0.4	3.1	0.6[a]	0.8[a]	5.2[b]
Roseomonas	Free living	0.0	0.0	0.0	3.0	0.0	0.3
Allorhizobium/Neorhizobium/ Pararhizobium/Rhizobium	Symbiont	0.0	1.2	3.0	3.2[a]	8.8[b]	9.0[b]
Bradyrhizobium	Symbiont	50.4	62.5	62.2	34.8[a]	54.3[b]	50.9[b]
Burkholderia-Caballeronia- Paraburkholderia	Symbiont	31.9	18.6	12.2	1.1	2.9	1.8
Cupriavidus	Symbiont	6.5	7.2	9.9	1.1	1.3	3.9
Devosia	Symbiont	0.0	0.0	0.0	5.8	3.0	3.1
Frankia	Symbiont	0.0	1.5	2.0	0.0	12.6	4.9
Herbaspirillum	Symbiont	0.9	1.0	0.0	0.0	0.0	0.0
Klebsiella	Symbiont	0.0	0.4	0.0	0.8	0.5	0.3
Mesorhizobium	Symbiont	0.0	0.7	0.0	3.9	9.3	12.6
Microvirga	Symbiont	0.0	0.0	0.0	4.2	3.4	3.3
Rhizomicrobium	Symbiont	2.9	3.6	2.5	0.0	0.0	0.2

b)

Genus	Moist Forest			Dry Forest		
	Pasture	YSF	OSF	Pasture	YSF	OSF
Albonectria	0	0	0.1	0	0	0
Botryosphaeria	69.6[a]	8.4[b]	5.6[b]	0	0	0
Calonectria	0.1[a]	5.7[b]	7.3[b]	0	0	6.3
Ceratobasidium	7.5	0.2	6.7	65.3	99.5	30.0
Cladosporium	2.5	0.5	0.1	2.4	0	3.2
Clonostachys	0.1	1.2	1.7	13.4	0	0
Cylindrocladiella	0	0.5	5.8	0	0	0
Cylindrocladium	0	0.4	0	0[a]	0[a]	44.9[b]
Fusarium	7.6	7.4	11.4	6.1	0	2.4
Ganoderma	0[a]	0.5[a]	9.1[b]	0	0	0
Gliocladiopsis	1.4[a]	30.7[b]	23.6[b]	0	0	0
Lasiodiplodia	0	0	1.0	0	0	0
Microdochium	1.3	1.5	0.1	8.6	0.2	0
Neonectria	0	0	0.7	0	0	0
Pestalotiopsis	2.1	1.9	0.3	0	0	13.4
Pseudocercospora	0.8	2.0	0.1	0	0.2	0
Rhizoctonia	0.8	0	0	4.2	0	0
Talaromyces	0	2.1	0.5	0	0	0
Trichoderma	4.5[a]	35.9[b]	24.7[b]	0	0	0
Xylaria	0.1	1.2	0.9	0	0	0

Table S4: PERMANOVA analysis of the response of soil microbial community assemblages to soil chemistry within a) Moist and b) Dry tropical forest landscapes, based on Hellinger-transformed relative abundances and Bray-Curtis dissimilarities. Target genera of Nitrogen fixing bacteria and plant pathogens were subset from the total bacterial and fungal communities (see Table S3). SS = successional stage, P = Phosphorus, Al = Aluminum, Ca = Calcium, Cu = Copper, Fe = Iron, K = Potassium, Mg = Magnesium, Mn = Molybdenum, Zn = Zinc.

a) Moist Forest

	**Total Bacteria**				**Total Fungi**			
Parameter	Df	R^2	F	*p*	Df	R^2	F	*p*
SS	2	0.14	3.33	**<0.01**	2	0.13	2.50	**<0.01**
pH	1	0.11	4.88	**<0.01**	1	0.05	1.96	**<0.01**
P	1	0.04	1.92	**<0.01**	1	0.04	1.63	**<0.01**
Al	1	0.04	1.80	**<0.05**	1	0.03	1.25	0.08
Ca	1	0.03	1.48	**<0.05**	1	0.03	1.24	0.06
Cu	1	0.03	1.39	0.06	1	0.04	1.42	**<0.05**
Fe	1	0.03	1.48	**<0.05**	1	0.03	1.18	0.10
K	1	0.03	1.20	0.18	1	0.02	0.98	0.54
Mg	1	0.03	1.32	0.08	1	0.03	1.14	0.16
Mn	1	0.03	1.19	0.17	1	0.03	1.03	0.39
Zn	1	0.02	0.99	0.45	1	0.02	0.96	0.58
Residual	22	0.47			22	0.55		

		**AMF**			**Nitrogen-fixing bacteria**			**Plant Fungal Pathogens**		
Parameter	Df	R^2	F	*p*	R^2	F	*p*	R^2	F	*p*
SS	2	0.21	4.69	**<0.01**	0.15	3.72	**<0.01**	0.18	3.55	**<0.01**
pH	1	0.06	2.68	**<0.01**	0.13	6.58	**<0.01**	0.04	1.44	0.09
P	1	0.04	1.76	**<0.05**	0.05	2.44	**<0.05**	0.03	1.06	0.37
Al	1	0.03	1.43	0.10	0.02	1.12	0.33	0.03	1.07	0.38
Ca	1	0.04	1.16	0.22	0.03	1.26	0.26	0.04	1.81	**<0.05**
Cu	1	0.03	1.63	**<0.05**	0.06	3.20	**<0.01**	0.03	1.09	0.32
Fe	1	0.02	1.19	0.24	0.02	1.00	0.46	0.03	1.13	0.29
K	1	0.02	0.92	0.59	0.04	1.86	0.07	0.02	0.74	0.79
Mg	1	0.03	1.12	0.29	0.03	1.74	0.07	0.03	1.38	0.12
Mn	1	0.03	1.58	**<0.05**	0.01	0.75	0.68	0.02	0.83	0.69
Zn	1	0.02	0.78	0.78	0.02	0.89	0.56	0.02	0.81	0.69
Residual	22	0.48			0.44			0.54		

b) Dry Forest

	Total Bacteria				Total Fungi			
Parameter	Df	R^2	F	*p*	Df	R^2	F	*p*
SS	2	0.20	4.57	**<0.01**	2	0.16	2.95	**<0.01**
pH	1	0.05	2.13	**<0.01**	1	0.03	0.92	0.71
P	1	0.03	1.38	0.07	1	0.03	1.08	0.23
Al	1	0.04	1.78	**<0.01**	1	0.03	0.95	0.60
Ca	1	0.03	1.40	**<0.05**	1	0.03	1.03	0.37
Cu	1	0.03	1.39	0.06	1	0.04	1.30	**0.04**
Fe	1	0.03	1.36	0.06	1	0.03	1.12	0.18
K	1	0.02	0.90	0.66	1	0.03	0.91	0.79
Mg	1	0.02	1.02	0.41	1	0.03	1.00	0.44
Mn	1	0.03	1.27	0.11	1	0.03	0.97	0.53
Zn		0.02	0.95	0.50	1	0.03	0.95	0.65
Residual	22	0.49			20	0.55		

	AMF				Nitrogen Fixing Bacteria			Plant Fungal Pathogens			
Parameter	Df	R^2	F	*p*	R^2	F	*p*	Df	R^2	F	*p*
SS	2	0.27	6.03	**<0.01**	0.22	5.33	**<0.01**	2	0.19	2.37	**0.01**
pH	1	0.03	1.38	0.09	0.08	3.87	**<0.01**	1	0.06	1.43	0.19
P	1	0.02	1.01	0.41	0.01	0.35	0.97	1	0.04	0.89	0.53
Al	1	0.02	0.83	0.72	0.03	1.34	0.22	1	0.03	0.73	0.67
Ca	1	0.02	0.95	0.48	0.03	1.40	0.19	1	0.04	0.90	0.49
Cu	1	0.04	1.95	**<0.01**	0.03	1.30	0.22	1	0.03	0.81	0.61
Fe	1	0.02	1.03	0.37	0.04	2.09	0.04	1	0.04	0.80	0.57
K	1	0.02	0.95	0.54	0.03	1.34	0.23	1	0.03	0.35	0.93
Mg	1	0.03	1.15	0.25	0.02	0.74	0.67	1	0.01	0.87	0.56
Mn	1	0.02	0.98	0.49	0.02	1.30	0.25	1	0.04	0.91	0.47
Zn	1	0.02	1.12	0.28	0.02	1.14	0.34	1	0.01	0.33	0.97
Residual	22	0.48			0.46			12	0.48		
